# Draft Genome Sequences of the Two Unrelated Macrolide-Resistant *Corynebacterium argentoratense* Strains CNM 463/05 and CNM 601/08, Isolated from Patients in the University Hospital of León, Spain

**DOI:** 10.1128/genomeA.00765-15

**Published:** 2015-07-09

**Authors:** Maria Isabel Fernández-Natal, Francisco Soriano, Alberto Acedo, Marta Hernandez, Andreas Tauch, David Rodríguez-Lázaro

**Affiliations:** a Department of Clinical Microbiology, Complejo Asistencial Universitario de León, New Caledonia, Spain; b Institute of Biomedicine (IBIOMED), León, Spain; c Public Health, School of Physiotherapy ONCE, Madrid, Spain; d AC Gen Reading Life, Valladolid, Spain; e Instituto Tecnológico Agrario de Castilla y León (Itacyl), Valladolid, Spain; f Institut für Genomforschung und Systembiologie, CeBiTec, Universität Bielefeld, Bielefeld, Germany; g Microbiology Section, Department of Biotechnology and Food Science, Faculty of Science, University of Burgos, Burgos, Spain

## Abstract

*Corynebacterium argentoratense* has been associated mainly with infections in the human respiratory tract. Genome sequencing of two unrelated clinical macrolide-resistant strains, CNM 463/05 and CNM 601/08, revealed the presence of the antibiotic resistance gene *erm*(X) allocated to a specific genomic region with 100% similarity to the widely distributed transposable element Tn*5432.*

## GENOME ANNOUNCEMENT

*Corynebacterium argentoratense* is a nonlipophilic, urease and nitrate reductase-negative, fermentative corynebacterial species producing acid from glucose but not from maltose and sucrose (1). It was initially isolated from patients suffering tonsillitis in Strasbourg, France (2, 3). Since the first isolation of *C. argentoratense*, it has been obtained from the respiratory tract of humans (4), blood cultures (5, 6), intravenous sites (7), ear (4), and conjunctival fornix (8) and from adenoid tissues associated with otitis media (9). The complete genome sequence of the type strain *C. argentoratense* IBS B10697^T^ (DSM 44202) comprises 2,031,902 bp, revealing so far the smallest sequenced genome of a corynebacterial species associated with humans (10). To attain additional genetic knowledge of this pathogen, particularly of clinically relevant antibiotic resistances, we sequenced the genomes of two unrelated clinical macrolide-resistant strains isolated from patients in the University Hospital of León, Spain. *C. argentoratense* strain CNM 463/05 was obtained in 2005 from a blood culture of a 3-year-old boy with upper respiratory tract infection, and strain CNM 601/08 was obtained in 2008 from a blood culture of an 85-year-old female with ischemic colitis.

Both *C. argentoratense* isolates were routinely grown at 37°C on blood agar. Genomic DNA was purified by using the ChargeSwitch gDNA mini bacteria kit (Invitrogen). Genome data were obtained as previously described (11) using an Ion Torrent PGM platform. The reads were collected by the Torrent Suite software version 4.0. The MIRA program version 3.4.0 (http://www.chevreux.org/projects_mira.html) was used for *de novo* assembly of the two genomes. Both genome sequences were annotated using the RAST genome annotation server (12).

Both *C. argentoratense* isolates showed similar genome sizes (approximately 2.02 Mbp) and similar numbers of protein-coding regions (approximately 1,900), with a slightly higher number (1.3%) in the genome of CNM 463/05. The draft genome sequences of both isolates revealed a high grade of similarity between them (>99.9%), as well as with the genome sequence of the type strain *C. argentoratense* DSM 44202 (>95.0%). BLAST was used for the search for antibiotic resistance genes in the Antibiotic Resistance Genes Database (13) and in published genome information of *Corynebacterium* strains. The antibiotic resistance gene *erm*(X) coding for macrolide-lincosamide-streptogramin B resistance was detected in the genome of both isolates and was allocated to a specific genomic region with 100% similarity to the composite transposable element Tn*5432*, initially found on the R-plasmid pTP10 of *Corynebacterium striatum* M82B (14, 15). This transposon is composed of two IS*1249* sequences, the *erm*(X) gene, and the partial transposase gene *tnpCX* (14). Transposon Tn*5432* is widely distributed in corynebacteria (1) and was also detected in bifidobacteria (16) and propionibacteria (17, 18), indicating that the horizontal transfer of *erm*(X) occurred between these actinobacterial phyla. Previous antimicrobial susceptibility assays demonstrated that *erm*(X) provides high resistance levels not only to erythromycin but also to azithromycin, josamycin, midecamycin, roxithromycin, spiramycin, tylosin, clindamycin, lincomycin, quinupristin, and pristinamycin IA (15, 17, 19). Resistance of clinical *C. argentoratense* isolates to these antimicrobials should be considered in the future when prescribing antibiotics for the treatment of infections with this commensal corynebacterium.

### Nucleotide sequence accession numbers. 

The whole-genome shotgun projects for the two *C. argentoratense* strains have been deposited in the GenBank database under the accession numbers JZEZ00000000 (CNM 463/05) and JZFA00000000 (CNM 601/08).
